# Behaviour change communication influences on food consumption behaviours and the demand for diverse nutritious foods in the Makoni District, Zimbabwe

**DOI:** 10.1371/journal.pone.0308012

**Published:** 2024-08-01

**Authors:** Delilah Takawira, Mthokozisi Kwazi Zuma, Xikombiso Gertrude Mbhenyane

**Affiliations:** 1 Division of Human Nutrition, Stellenbosch University Faculty of Medicine and Health Sciences, Tygerberg, Cape Town, South Africa; 2 Food and Agriculture Organization Zimbabwe, Harare, Zimbabwe; Federal University of Agriculture Abeokuta, NIGERIA

## Abstract

This study evaluated the effectiveness of nutrition behavioural change communication interventions and food consumption behaviours and demand for nutritious foods in Makoni district, Zimbabwe. The study employed an observational and cross-sectional design using mixed—methods. The population was smallholder farmers’ households with children six to twenty-four months old. Secondary data was obtained from the main intervention reports. The total sample size of this project comprised of five personnel participating in the implementation of the intervention for key informant interviews; forty participants for the in-depth interviews; and a total of 81 participants for eight focus group discussions. Participants indicated that the Livelihoods and Food Security Programme intervention successfully increased their nutrition knowledge, enhanced their ability to diversify crop production, and improved their access to varied foods, including some new crops. Local markets had little influence on the demand for nutritious foods by the intervention population. The interventions were effective in stimulating demand for diverse and nutritious foods in Makoni District.

## 1. Introduction

Malnutrition, particularly child stunting, has long-lasting effects on the overall growth and development of affected children [1]. A stunted child is more likely to contract non-communicable diseases and lessened cognitive capacity. This makes them more prone to perform poorly at school and have up to 10% reduced earning capacity in adulthood compared to their non-stunted counterparts [2]. Nutrition-sensitive agriculture interventions are essential in addressing malnutrition in low- and medium-income countries [3].

Agriculture is the source of all nutrients that sustain human life [4]. Increased production and farming of a variety of crops and livestock, especially small stock, is vital in determining what people eat, particularly amongst poor rural households who rely on their own local production for consumption. It is also in these very areas where the prevalence of malnutrition amongst women and children is high. Available evidence has shown that nutrition behaviour change communication interventions are an important instrument for turning increased agriculture production into improved nutrition [3].

Several behaviour communication change theories have been proposed and can be used to influence behaviour change effectively [5]. A review of health and nutrition behaviour change interventions focusing on the linkages between behaviour change theories and techniques identified that most interventions were designed without any underlying behaviour change framework or theory [6–9]. Some of the common health behaviour change techniques include cognitive or mental, behavioural, social, sensory and social [6, 7, 9].

Similarly, several methods can be used to assess the nutritional adequacy of diets, including the use of food consumption indices. There is, however, a paucity of knowledge of the effectiveness of such interventions in farming communities, including how to create demand for nutritious foods produced. The effectiveness of interventions was investigated through an evaluation of the Livelihoods and Food Security Programme (LFSP) impact [10]. The LFSP was an agriculture value chain intervention implemented in eight districts across three provinces in Zimbabwe. One of these districts was Makoni in the eastern province of Manicaland. This intervention was created for Improved Nutrition for Sustainable Production and Increased Resilience for Economic Growth (INSPIRE), led by Goal Zimbabwe [11].

The LFSP was a nutrition sensitive-agriculture intervention, aimed, among other things, at contributing to reducing stunting in Makoni district. An estimated 34% of children under the age of five in the Makoni district were stunted [12]. The intervention aimed to achieve its objective through increasing the production and consumption of diverse and nutritious foods, focusing on households as well as women of childbearing age and children aged 6 to 24 months. In addition, positive nutrition and health behaviours, such as household hygiene, hand washing, exclusive breastfeeding for children aged 0 to 6 months, optimum complementary feeding for children aged 6 to 23 months and prevention of childhood illnesses were promoted. The first 1000 days approach was used for targeting the nutrition intervention.

The LFSP intervention targeted communal smallholder farmers and their families to improve food, nutrition, and income security within these households by promoting viable agriculture value chains of high-value crops, such as sugar beans (including high iron and zinc biofortified beans), green maize (including a biofortified Vitamin A orange maize variety), paprika, other horticultural crops and small livestock such as broiler and layer chickens, beef fattening and goat production [13]. Climate-smart agriculture approaches were promoted to curb the negative effects of unpredictable weather and rainfall patterns on agricultural productivity.

The INSPIRE intervention adopted an approach called Nutrition Impact and Positive Practice (NIPP), which is essentially a peer-to-peer, positive deviance behaviour communication change strategy. Positive Deviance is based on the observation that in every community there are certain individuals or groups whose uncommon behaviour and strategies enable them to find better solutions to problems than their peers. The NIPP addressed multiple causes of malnutrition in a holistic manner, focusing on dietary diversity, water and sanitation, and hygiene promotion [10]. Nutrition education and training were also provided to agriculture extension officers, and they passed this information on to both men and women farmers as they interfaced with them during agriculture extension activities. These messages were centred on promoting diversified production for household consumption using cropping methods like conservation agriculture, crop rotation, and intercropping [10].

This study aimed to evaluate the effectiveness of nutrition behaviour communication change by the LFSP (hereafter referred to as the intervention) in increasing the demand for diverse and nutritious foods by smallholder farming households in Zimbabwe’s Makoni District, and the influence of social, cultural and market factors on this increased demand.

## 2. Materials and methods

### 2.1 Study design

The study adopted a cross-sectional design, using mixed-methods. The qualitative approach followed a phenomenological approach with ethnographic consideration [14]. Primary data from this study was compared to intervention baseline secondary data of selected indicators. Quantitative data was collected to compare the outcome variables at the beginning of the main intervention and at the time of this study.

### 2.2 Study population

The population studied was smallholder farming households living in the Makoni rural District of the Manicaland Province, East of Zimbabwe. This study focused on households with men, women of childbearing age, and children aged six to twenty-four months who participated in the LFSP-INSPIRE intervention. This study population was spread over two main agroecological regions: 2b and 3 –reaching a total of approximately 18,000 smallholder farming households. The intervention covered 30 out of the 35 administrative wards in the district. This study area was stratified into two clusters according to the two main AERs from the main study. This study was conducted from 1 November 2018 to 30 April 2019.

### 2.3 Sampling and data collection procedures

Secondary data was obtained from the main intervention reports over the five-year implementation period from 2013 to 2017. The total sample size of this project comprised of five personnel participating in the implementation of the main intervention for key informant interviews; forty participants for the in-depth interviews; and a total of 81 participants for eight focus group discussions. The main researcher assisted by two field workers conducted the interviews. The main researcher is a Nutritionist and at the time worked for Food and Agriculture Organisation in Zimbabwe. The research assistants were either an Agricultural extension officer or Health promotion officer. The research assistants were trained by the researcher and one of the consultants from Food and Agriculture Organisation in Zimbabwe.

All instruments were piloted before data was collected. The primary objective of the pilot study was to pre-test all data collection tools before they were finalised and printed. All data collection tools were administered during the pilot study:

- The Guide: two Focus Group Discussions (FGD) using women and men participants from nutrition behaviour communication change groups–ENIPPA Circles. The researcher led the discussions while the research assistant recorded the participants’ responses from the discussion.
- The questionnaire: two Key Informant Interviews were conducted with participants who were intervention officers were conducted by the researcher and research assistants.
- Household Food Insecurity Access Scale (HFIAS) questionnaire and 24-hour diet recall for Household dietary diversity score (HDDS); Mean dietary diversity (MDD); and MDD-Women administered by the research and research assistants.

#### 2.3.1 Desk review study of intervention documents

The investigation started with a desk review study of intervention programme documents. The primary aim was to establish the intervention baseline status of programme participants and the methods used by the intervention to stimulate demand for diverse and nutritious foods. Documents reviewed included the programme proposal, baseline survey report, ENNIPA methodology document, and quarterly and annual programme reports. A desk review study checklist guided the secondary review.

#### 2.3.2 Qualitative data

*2*.*3*.*2*.*1 Key informant interviews with programme personnel*. Five key informant interviews were conducted with intervention personnel who supported the programme in the district. These were either Non-Governmental Organisation (NGO) staff or government officers working in the district who were actively involved in the programme. Key informants were selected using purposive sampling. The objective of this data collection method was to verify the actual nutrition behaviour communication change activities [15] implemented by intervention and comprehend how well these were done, the challenges that were faced, and officers’ perceptions about the intervention and how well it managed to increase the demand for nutritious foods among households including women and children.

*2*.*3*.*2*.*2 Focus group discussions with programme participants*. The FGDs were used as an approach to engage participants and understand their perceived benefits from the intervention as far as demand for nutritious foods–assessed through food consumption patterns–as well as other aspects of nutrition behaviour change in this study. The discussion also queried the factors affecting the adoption of the promoted practices and behaviours. FGDs were conducted with a separate group of participants to the individuals who participated in the in-depth interviews. FDG participants were, however, all intervention beneficiaries residing in the same communities and environments. Maximum variation purposive sampling was used to select groups of women and men from the 30 intervention wards in Makoni District. The study population was split into two clusters by agroecological region: 2b and 3. In each cluster, mixed groups were selected to participate in the FGDs as there were no separate male and female behaviour communication change groups in the intervention. In all but one case, the ratio of male to female participants was approximately 1:3. A total of 81 participants (58 females and 23 males) participated in the FDGs. The FDGs were eight with between eight and 13 participants. See Table 1 below:

**Table 1:** Sample selection of focus group discussions.

AEZ	Area / Ward	Name of group	Number of People in the Focus group (n)		
			Female	Male	Total
**2b**	1	Village A	10	3	13
		Village B	8	5	13
	2	Village C	7	3	10
		Village D	5	3	8
**3**	3	Village E	7	3	10
		Village F	6	2	8
	4	Village G	9	0	9
		Village H	6	4	10
**TOTAL**			**58**	**23**	**81**

AEZ: agroecological zone

#### 2.3.3 Quantitative data

*2*.*3*.*3*.*1 In-depth interviews with adults participating in the programme*. Forty in-depth interviews were conducted with 30 women and ten men selected through purposive random sampling from the two study clusters. The objectives were to obtain socio-demographic parameters of households, establish the households’ food security status, household dietary diversity, and the dietary diversity of women of childbearing age (15 to 49 years) and children aged six to 24 months. These interviews also sought to identify any consumption pattern changes because of the intervention, participants’ perceptions about the intervention’s effectiveness, as well as barriers and enablers for optimum food consumption. The *Shona* translations of in-depth interview questionnaires were used. The principal investigator, with the help of the research assistant, administered the questionnaires.

After random selection of interviewees and ascertaining that women participant had children aged 6–23 months or at most 36 months in their care, an interview date was set. All interviews were conducted at the participant’s household. Each interview started with establishing rapport with the participant, asking about general issues and ensuring the participant is settled and not anxious. An explanation of the study objectives was given, together with further details about the research process. Participants were assured of confidentiality and an explanation was given of how the information collected would be used as outlined on the study information sheet, which was also translated into the local language *Shona*. Participants could ask questions to clarify any issues before being asked to sign the informed consent form. All the selected participants agreed to participate in the study. Each interview took approximately one hour and all 20 interviews in a cluster were covered in two days.

Two data collection questionnaires were developed for male and female interviewees, and these were translated into *Shona* before administration. Quantitative data indicators collected were: household food insecurity access scale (HFIAS), household dietary diversity score (HDDS), household food consumption score (HFCS), minimum dietary diversity for women (MDD-W) and for children aged six to 24 months (MDD). Other quantitative data collected through the questionnaire included data on household livelihood sources, household food production, and household demographics. Validated instruments for collecting quantitative data were used for the HFIAS, HDDS, HFCS, MDD-W and MDD indicators to ensure the validity of the assessment.

*2*.*3*.*3*.*2 Analysis of qualitative and quantitative data*. Qualitative data were analysed using NVivo version 13 –a statistical package for qualitative data analysis. The analysis started by carefully typing out all responses from the questionnaires into Microsoft Word. The data were then imported into NVivo where common themes were identified and grouped. Frequencies of occurrence of common themes were identified, and the data were reported quantitatively.

Quantitative in-depth interview data was analysed using the IBM Statistical Package for Social Sciences (SPSS), version 24. Data analysis started with a review of all the questionnaires to check for obvious mistakes, completeness and identification of any data recording errors. All the quantitative questionnaires were pre-coded during development. The next step therefore involved data entry into Microsoft Excel whereby data was coded to identify the levels or scales of measurement as nominal, ordinal, interval or ratio. The data was then exported into SPSS for analysis. Descriptive data analysis then followed. Frequency tables were used to indicate the number and percentage of participants in each category, histograms showed the percentage of all participants falling into a category.

Data were summarised using descriptive analysis. Medians or means were used as the measure of central tendency for numerical variables. The relationship between nominal variables–comparing the frequency of different responses–was compared using contingency tables and the Chi-square test. Data were disaggregated by gender and AER during analysis. No differences were identified between these categories and as such the data were reported together.

#### 2.3.4 Ethical approval

*2*.*3*.*4*.*1 Ethical review committee*. The protocol was approved in writing by the Health Research and Ethics Committee, Faculty of Medicine and Health Sciences, Stellenbosch University (reference number SI/16/04/067).

*2*.*3*.*4*.*2 Authority to conduct the study*. The researcher sought formal permission to conduct the study from the team leader of the non- NGO consortium running the intervention in the Makoni district. This consortium already had authority from the provincial and district administrative leaders to implement the intervention in the district.

*2*.*3*.*4*.*3 Informed consent*. Participation in the study was voluntary for all participants following an informed written consent. All participants were invited to participate at their free will. On the day of the appointment, the study objectives were clearly explained, and confidentiality issues clarified. Participants could ask questions to seek any further clarifications, and these were responded to clearly and detailed. The informed consent, which was translated into *Shona* prior to data collection was clearly read to the participants. Participants were each allowed to decide whether to participate and to sign the consent form before the interviews or discussions began. Even after agreeing to participate in the study and signing the consent forms, participants were still allowed to decide to stop the interview or leave the discussions at any given time. The study adhered to the principles of The Declaration of Helsinki 2013.

*2*.*3*.*4*.*4 Participant confidentiality*. Participant confidentiality was observed and respected before, during and after data collection. None of the participants’ names were recorded on the questionnaires and the participants were assured that their names would not be used at all, and that the information collected from them would only be used for the purposes of the study together with information from other participants.

## 3. Results and discussion

The study revealed that the behavioural communication change approach was effective in stimulating demand for nutritious foods by the households in this study. The findings are presented, interpreted and discussed under qualitative and quantitative data categories with the following subheadings integrated: behaviour communication change activities implemented, intervention effectiveness in stimulating demand for nutritious foods, and factors influencing the demand for nutritious foods.

### 3.1 Qualitative data

Key themes that emanated from key informant and focus group discussions include participants’ understanding of a balanced diet, participants’ understanding of the consequences of not eating a variety of foods/balanced diet, key lessons about healthy eating from ENIPPA, determinants of children’s and household food consumption patterns, changes in food consumption as a result of the intervention, major challenges to optimum household food consumption and sustainability of acquired healthy eating practises. These themes are integrated in the reporting and discussion that follows.

The results of key informant interviews with intervention staff confirmed that the interventions implemented many activities to stimulate and promote an increase in the demand for varied and nutritious diets. As reported by the informants, techniques used in the intervention included the use of songs, drama, poems, discussions, home visits, food preparation demonstrations, food fairs, and demonstrations for establishing micro-gardens (backyard home gardens) and low-input gardens. Home gardens have been identified as one of the possible ways of producing food and offer great solutions to some of the issues surrounding poverty alleviation and improving food security in rural areas [16]. The informants reported how women were supported as groups. Activities of precision livestock farming are the compromise for environmental, economic and socially sustainable production.

Support for the groups included small livestock production focusing on chickens in addition to gardens as a way of increasing access to diversified foods, particularly those observed to be lacking in their diet, such as meat and animal source foods. Small livestock farming during the intervention was, however, minimal. Informants also reported how graduation ceremonies were used as motivation to encourage people to join the groups–they gave participants a sense of accomplishment. Hedge [17] asserts that livestock is significantly contributing to the livelihood and food security of more than a billion people in different parts of the world. A review of the role of cattle livestock farming was done to determine if it will support the process of decision-making of farmers. In addition, they should change their role on the farm and their management view and make possible the traceability of products and the control of the quality of products and the animals’ living conditions, as required by policymakers and stakeholders [18].

#### 3.1.1 Intervention effectiveness in stimulating demand for nutritious foods

Key informants’ perceptions of the intervention, as far as stimulating the demand for nutritious foods, indicated that the intervention promoted micro-gardens. Intervention participants were introduced to new crops that helped them increase the availability and access to a wide range of foods. Adenle, Wedig and Azade argue that public-sector research and international organisations can significantly contribute to the adoption of evidence-based policies that support context-specific combinations of low- and high-tech approaches by small-scale farmers [19]. The intervention increased participants’ appreciation for dietary diversification which helped to increase the uptake of the promoted methodologies. The informants also reported that the intervention managed to promote the consumption of balanced diets, increased meal frequency and increased dietary diversity for women and children aged six to twenty-four months. These findings are in line with many studies that show how increasing access to a wide range of foods complemented with nutrition education and behaviour communication change is essential to increasing demand for nutritious foods [20–23]. The key informants indicated that the intervention provided support to participants through the provision of basic vegetable seeds (for example spinach, carrots, beetroot and peas) to start up the gardens. These seed packs included bio-fortified crop varieties for Vitamin A orange maize and high iron and zinc beans. Other support included facilitating groups’ activities such as arranging food preparation demonstrations, food fairs, and conducting household support visits. Literature confirms that support towards increasing the production of varied foods is essential for stimulating the demand for nutritious foods [24–26].

Findings from the key informant interviews were confirmed by the participants engaged through the in-depth interviews and FGDs. These household participants confirmed that they were consuming many new foods that they never consumed before the intervention. They also indicated that through the intervention, they learned about healthy eating, using natural pesticides for sustainable crop production, and using different recipes for meal preparation. All these activities were done to stimulate the demand for diverse, nutritious foods. FGD participants also confirmed that they relied mostly on their production for household consumption [25, 26].

The results of this study are broadly consistent with findings from other studies [20, 27] that show that agriculture production coupled with nutrition education and/or nutrition behaviour communication change is essential for stimulating the demand for nutritious foods and promoting diversified consumption at both household levels and for women and children. Unlike most of the studies documented in the literature, this study used a mixed methods approach which allowed soliciting of participants’ views, perceptions and understanding of the intervention’s efforts towards stimulating the demand for nutritious foods. For instance, while key informants and participants in this study largely agreed that the intervention implemented many activities to stimulate demand for nutritious foods, they also indicated that the scale of the interventions was too small to have a meaningful impact at the district population level. Findings also showed that not all intervention participants received the initial seed packs and although they were convinced that there was a need to diversify their diets and fully appreciated this, they reported that they did not have the necessary resources to continue producing the required crops and livestock including the lack of income to buy the required inputs.

Participants in this study said they still needed further support to be able to sustain the adopted behaviours. Underlying the apparent lack of dietary diversity among most rural households in the Makoni District and Zimbabwe in general is deep-rooted poverty emanating mostly from the failing national economy. The unpredictable weather changes because of climate change affecting many smallholder farmers’ abilities to produce adequate food for their household consumption further intensifies the challenges [28–33]. Other findings pointed to the need for more intervention staff to be able to fully support the farmers.

This study revealed that the intervention activities implemented were effective in stimulating the demand for a wide variety of foods among participants’ households as well as among women and children. In addition to the variety of interventions and activities implemented to stimulate demand for varied and nutritious foods, findings from the situational analysis using the intervention’s primary data and the empirical in-depth interviews in this study, indicated a marked improvement in the demand and consumption of varied foods.

### 3.2 Quantitative data

#### 3.2.1 Food security

**Fig 1:**
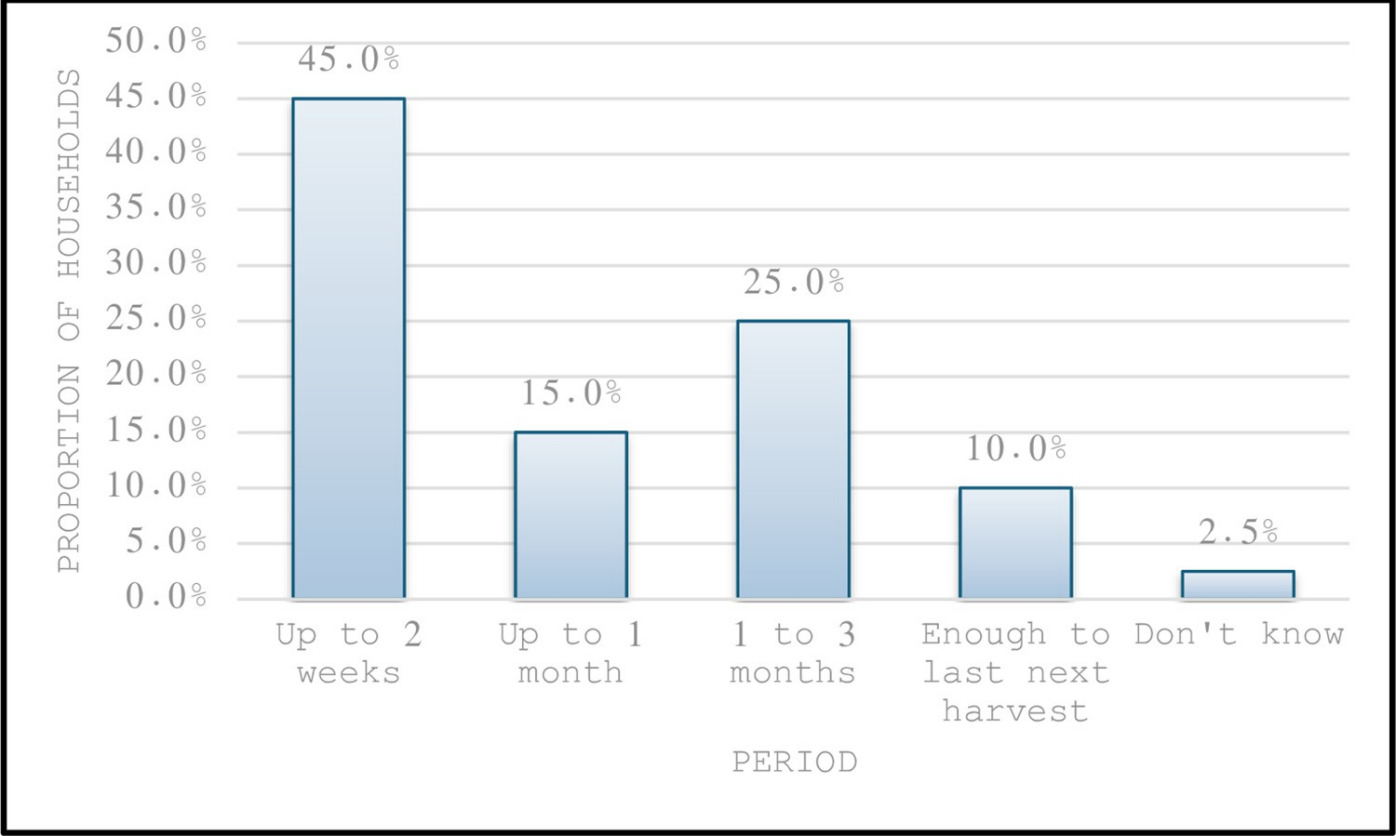
Household with cereal lasting for the various time periods.

The most commonly consumed cereal in the households was reported to be maize. Most households (97.5%) had maize stocks for consumption lasting 2 weeks or less (45%). Only 25% of households had stocks that could last up to 3 months. This question was asked as a proxy for household food security status. See Fig 1. The intervention baseline survey reported that approximately 90% of households (92.5% male headed and 86.4% female headed) had little or no hunger, as this study observed. In this study, 70% experienced little or no hunger. According to the Zimbabwe Food and Nutrition Council report, 35.83% of the population was food insecure in 2019/2020 with 64.17% experiencing little or no hunger [12]. Thus, Mankoni district, which is rural still has high levels of food insecurity.

#### 3.2.2 Dietary diversity

**Fig 2:**
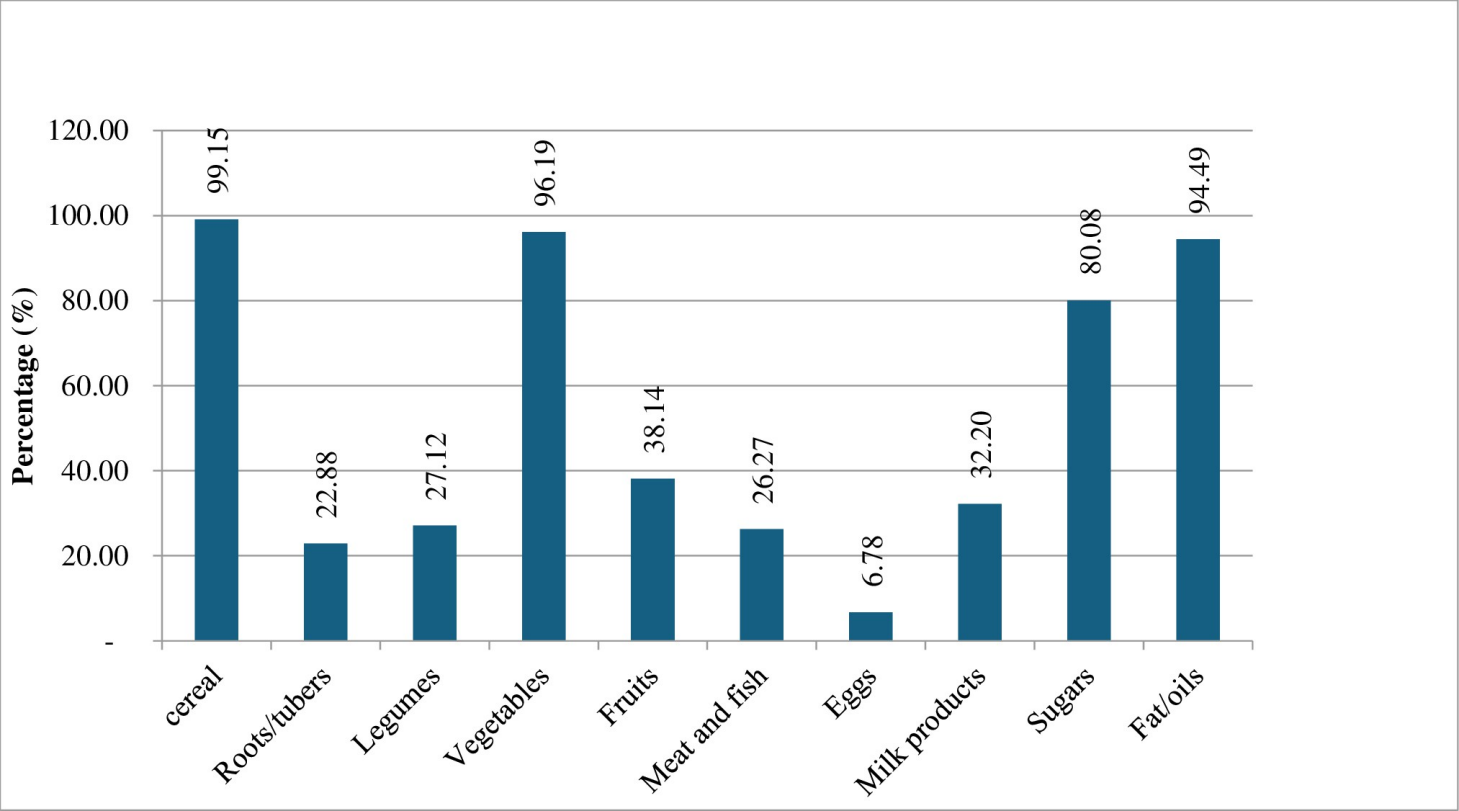
Consumption of various food groups by households in this study.

Although assessed through different means, household dietary diversity was better in this study than the baseline secondary data (before the intervention). Using HDDS, only 26.7% of households were consuming five food groups in the intervention and less than 24% were consuming six or more food groups in this study. The most consumed foods–consumed by more than 80% of the households–were cereals, vegetables, sugar and fats/oils for this project. Eggs were consumed by only 6% of the households, while meat and fish were consumed by 26.7% of the households, and legumes by 27.12% of the households. Fruits were consumed by 38.4% of the study population and milk and milk products by 32.2% (see Fig 2).

**Fig 3:**
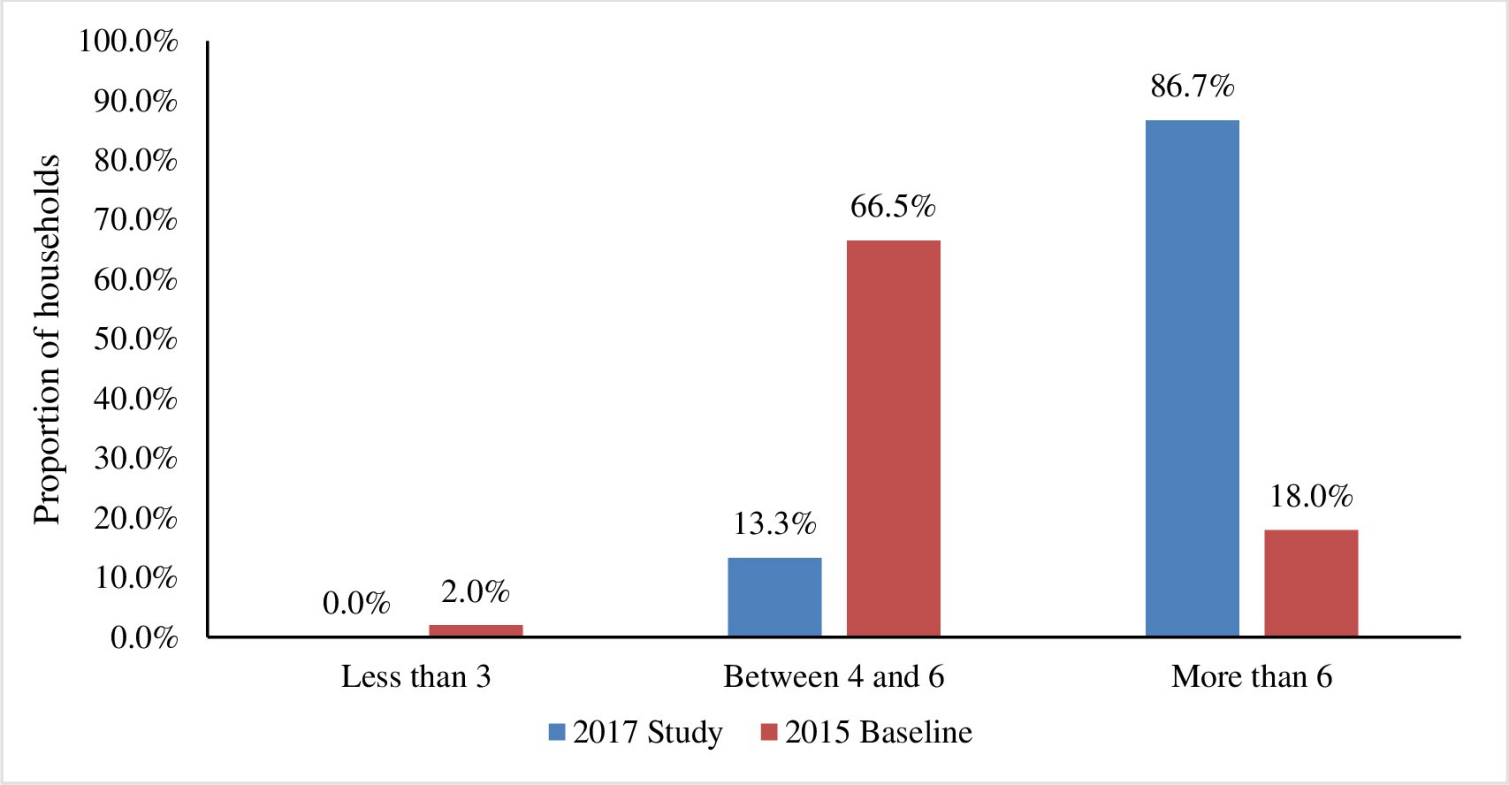
Household dietary diversity score by group categories in this study.

**Fig 4:**
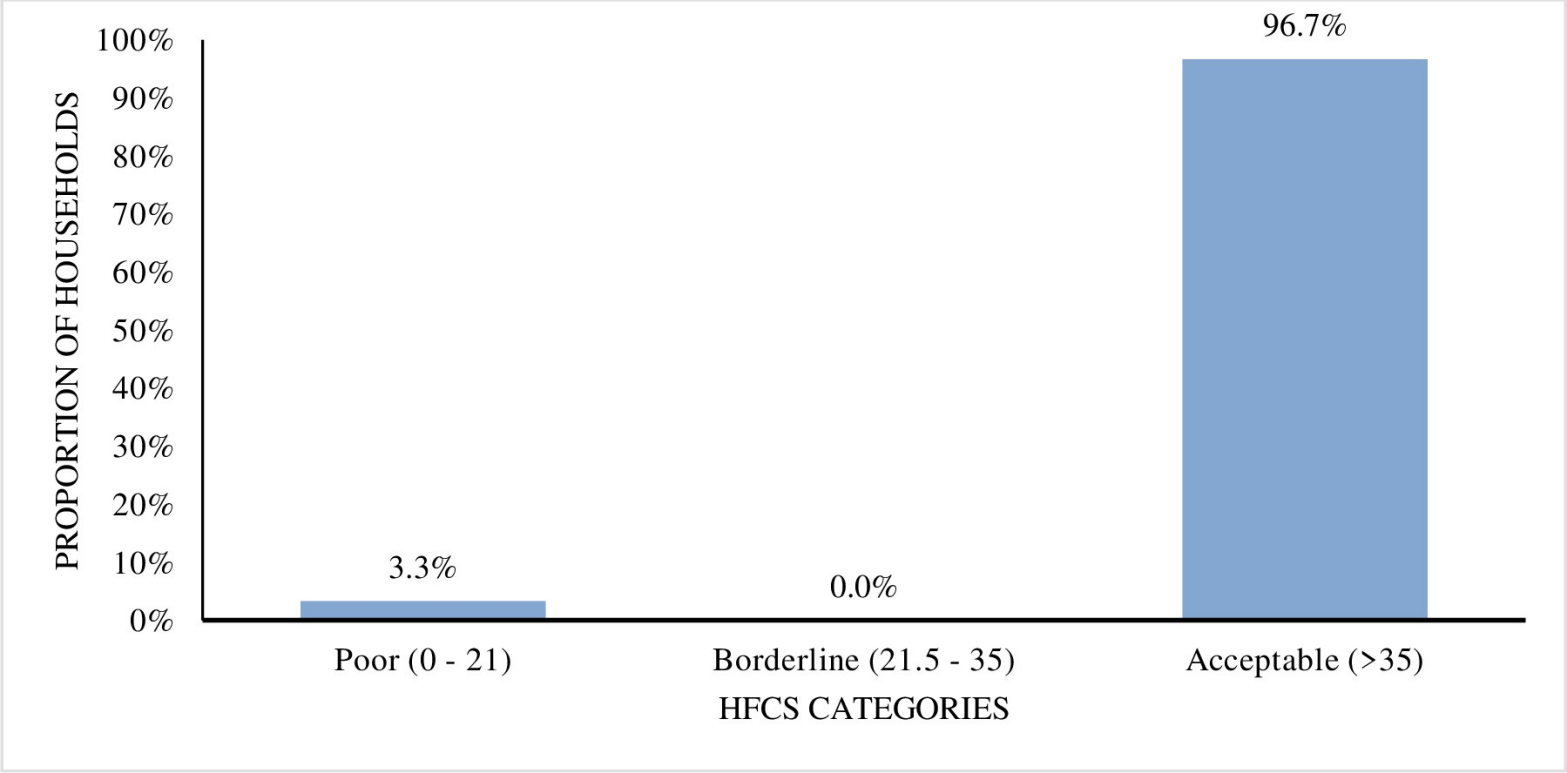
Household food consumption score by category in this study.

In comparison, the study findings from in-depth interviews with intervention participants conducted two years after the baseline revealed that 86.7% of participants’ households consumed foods from more than six food groups using the HDDS, and 96.7% of households had an acceptable HFCS (see Figs 3 and 4). This study–which used the more sensitive food and nutrition security indicators–indicated that participants fared better with the results of this study compared to the intervention baseline data. The HDDS grouping categories were, less than 3 (poor), between 4 and 6 (medium) and more than 6 (high), Fig 3.

**Fig 5:**
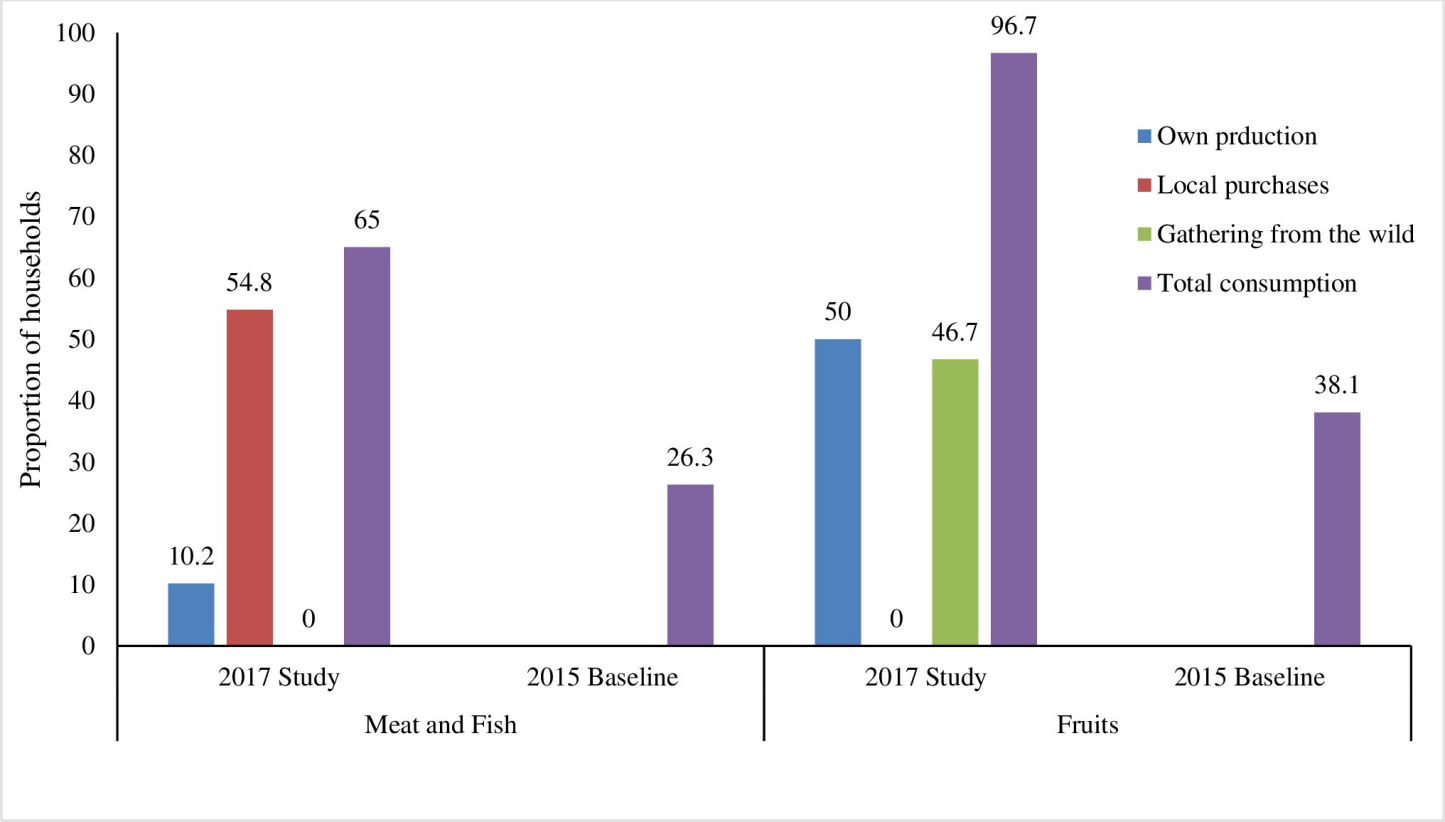
Household consumption of meat and fruit and their sources.

In addition, at the baseline of the intervention, households were consuming fewer food groups than observed through in-depth interviews in this study. Similarly, consumption of meat, fish, and fruit was higher in this study than at the intervention baseline. Fig 5 shows that 65% of households were consuming meat and fish in this study compared to 26.3% at baseline and 96.7% of households were consuming fruits after the intervention compared to 38.1% at baseline.

**Fig 6:**
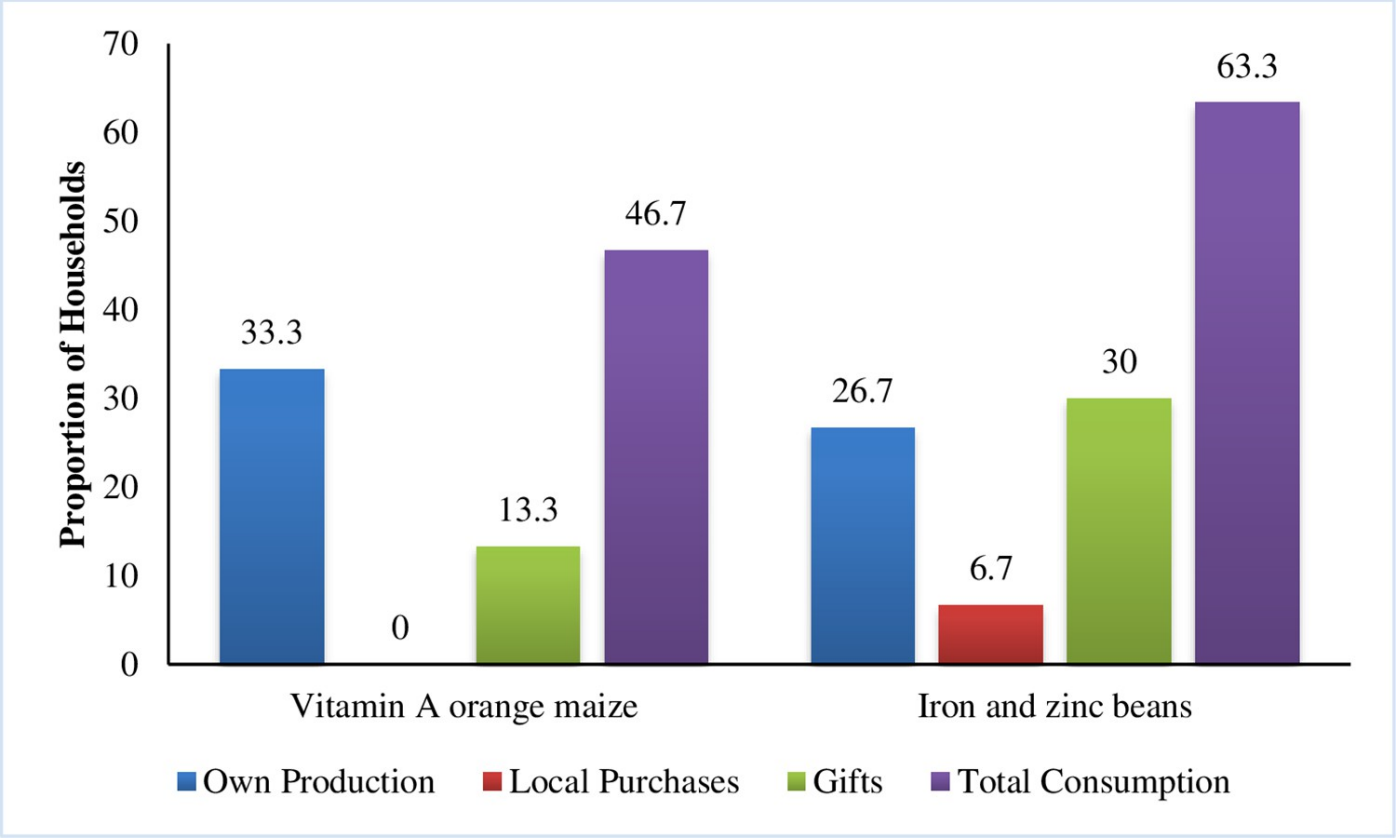
Household consumption of bio-fortified crops and sources of the crops.

Households in this study were consuming biofortified crops–vitamin A orange maize and beans, as depicted in Fig 6. Most of this food came from the participants’ own production. Bio-fortification is one proven agricultural intervention with proven impacts on nutrition [34]. Several studies have proved that regular consumption of bio-fortified crops can improve micronutrient stores in the body, reverse micronutrient deficiencies, and increase resistance to infections [35–39].

Following the intervention all households were growing many vegetables including green leafy vegetables, tomatoes, onions, green beans, butternuts, carrots, and spinach. Crop production is synonymous with increased consumption of the crops grown, especially when accompanied by nutrition education and behaviour communication change [40].

**Fig 7:**
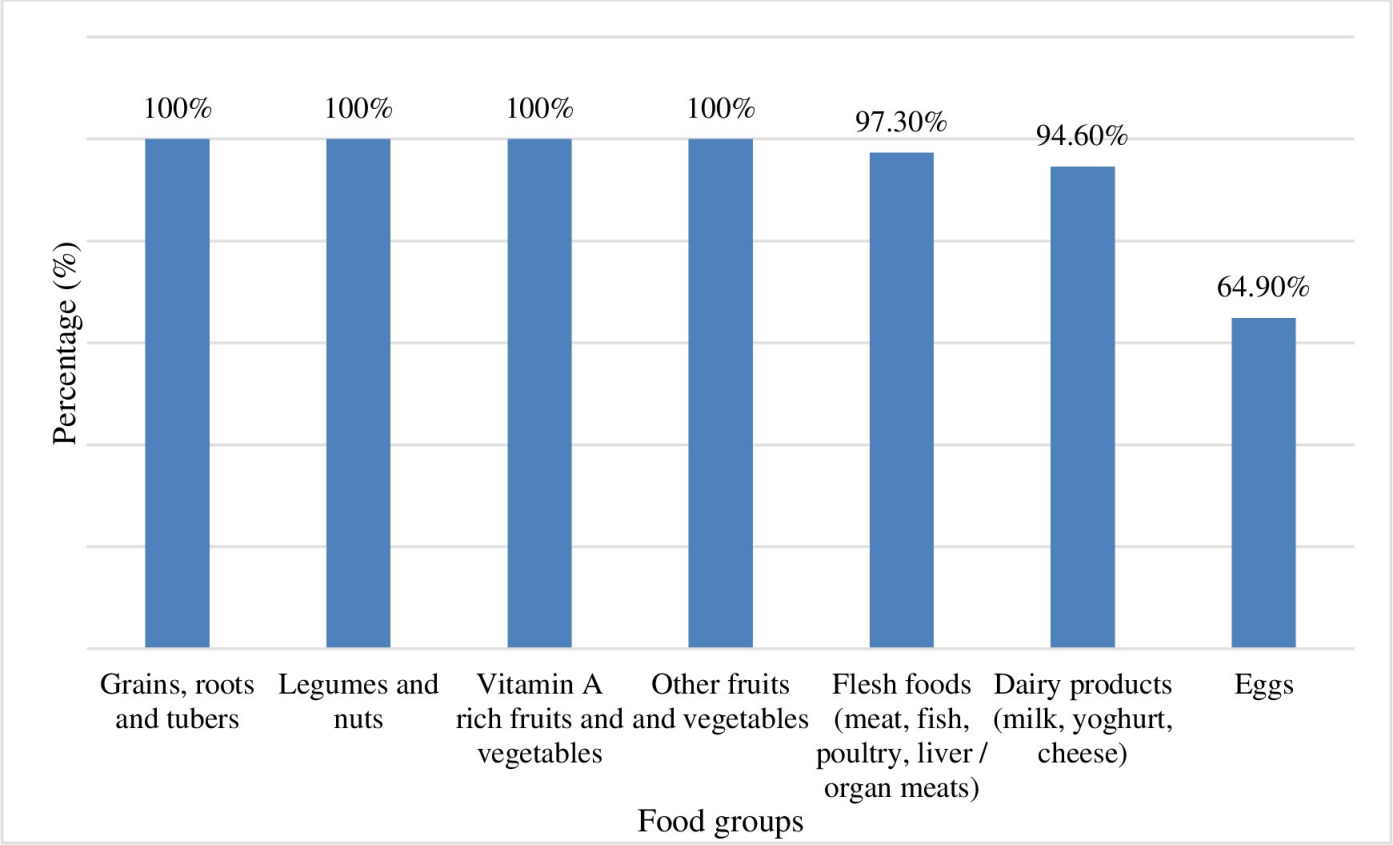
Various food consumption by children aged six to 24 months in this study.

This study demonstrated that the behaviour change methodology employed effectively improved child feeding practices within the two years in the Makoni District. Only 12% of children aged six to 24 months were receiving a minimum dietary diversity before the intervention. When assessed during this study, all children aged six to 24 months (100%) included in the sample were consuming at least four of the seven food groups included in the calculation of this indicator. Children consuming a diversified diet are more likely to meet their nutrient requirements and be well-nourished [41]. As indicated in Fig 7, most children were consuming the following food groups: grains, roots and tubers. This finding concurs with other studies showing that interventions that combine diversified agricultural production with nutrition education and behaviour communication change are more likely to be effective in improving child-feeding practices [42].

The intervention directly targeted the four-star diet for children aged six to 24 months as a crucial behaviour to promote [43]. Adequate dietary intake and good health (absence of disease) are directly associated with good nutritional status. The absence of these two is the immediate cause of malnutrition [44]. The absence of adequate dietary intake, i.e. a child receiving a diet that is deficient in key food groups, and hence nutrients, is more likely to be ill, which in turn, affects health and causes malnutrition.

This study’s findings, however, also picked some negative feeding practices which if continued unchecked, could cause problems with overweight and obesity among children. In addition to consuming healthy foods, a significant proportion of children were also consuming high-calorie, less nutritious foods such as sugary drinks/sodas, pastries, biscuits and sweets, tea and coffee. Zimbabwe, like any other low-to-medium income country, is experiencing the double burden of malnutrition, with a growing problem of overweight and obesity, particularly among women and children. Unfortunately, consistent with findings from other countries, nutrition interventions continue to focus on undernutrition instead of malnutrition [45]. None of the ten behaviours promoted by the intervention focused on overweight and obesity, although it can be argued that the four-star diet promotion focused on healthy food options and so did optimum complementary feeding promotion, which includes both food quality and quantity.

**Fig 8:**
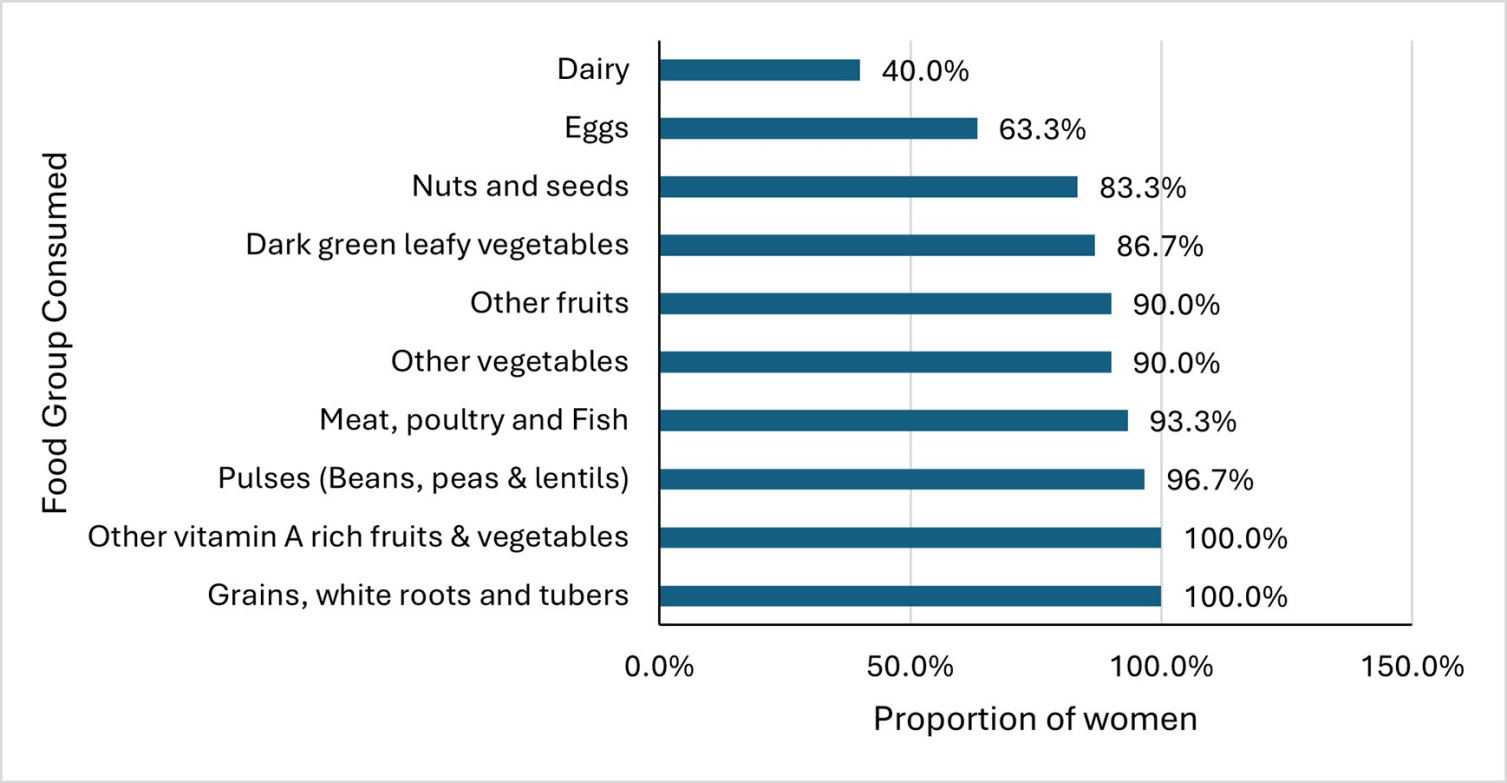
Consumption of various food groups by women aged 15 to 49 years old in this study.

This study also showed that women of childbearing age (15 to 49 years old) were consuming diversified diets. This indicator was unfortunately not assessed at the onset of the intervention. However, 93.3% of women in this study were consuming foods from at least five of the 10 food groups. The MDD-W estimates the likelihood of women meeting their macro and micronutrient requirements from the diet [46]. Women of childbearing age were at risk of micronutrient malnutrition, especially deficiencies of iron, calcium and Vitamin A. The MDD-W can be used as a population-level indicator for assessing women’s dietary diversity. More than 80% of women were consuming eight of the ten food groups two years after the intervention started in Makoni District, except for the dairy and eggs food groups which were consumed by 40% and 63.3%, respectively (Fig 8).

These results for women and children show that eggs were not that widely consumed by the participants. This observation could have also been caused by a reported taboo forbidding pregnant women from consuming eggs and avocados [47]. The women’s dietary diversity was not assessed at baseline, so the study was not able to compare the situation during this study to what was happening before the intervention. However, if the children’s feeding patterns are anything to go by, then one can deduce and say they were as worse off as the children’s dietary patterns before the intervention. The intervention methodology also included the promotion of behaviour about women’s dietary diversity on consumption of iron-rich foods. Other targeted behaviours also associated with the improvements seen included the establishment of household micro-gardens and the preservation of vegetables by solar drying.

The production of animal-source foods was not among the ten behaviours promoted by the intervention. It may also explain why egg consumption was slightly low for both women and children in this study. Consistent with this, results show that 54.8% of meat and fish consumed by households was purchased from local markets rather than their own production, whereas crop consumption shows that most crops were from their own production. In addition to the intervention, many other agriculture value chains including poultry, goats, and eggs were promoted. However, these interventions targeted selected farmers with the potential to produce and market their produce. It appears the animal food sources were being bought from these farmers as the rapid market assessment showed that most people in Makoni District bought their food from other villagers producing excess rather than from formal markets.

### 3.3 Intervention effectiveness in stimulating demand for nutritious foods

Complementary to the quantitative findings, both key informants and participants in this study reported that the intervention was effective in promoting the consumption of diversified diets. Participants in this study were able to define a balanced diet and could link child stunting to poor food consumption. Participants indicated that through the intervention they learned about disease prevention, preparing nutritious meals, food and nutrients, and the production of nutritious foods. Findings from the FGDs indicated that the intervention helped participants increase their knowledge of healthy eating, increased their ability to grow a wide variety of crops for improved nutrition, and increased their access to many foods including some new crops they had never included in their diets. Participants also reported improved knowledge of food preparation and improved child feeding with increased diversity through enriching maize-meal porridge–which is what children are mostly fed. Through the intervention sessions, participants were now able to stay healthy, eat diversified diets, cook various foods using different recipes, produce their foods cheaply, and work hard with their own hands.

### 3.4 Factors influencing the demand for nutritious foods

This study has shown that agriculture production is both a determining factor and an indicator of the demand for foods consumed by households. Before the intervention in Makoni District, diversified crop production was low–reported as 56% of households growing one type of crop and 20% and 16% of households growing two and three types, respectively. This was associated with reduced dietary diversity for both households and children as highlighted previously. The findings of this study revealed that agriculture production within the intervention was influenced by several factors. Key informants reported that the seed distributed to households to start micro-gardens was not adequate and many intervention participants did not receive the seed packs. It would go without saying that the participants who did not receive seed packs may not have produced as much as those who received the seed. If the promotion of diversified agricultural production for improved consumption and nutrition was important to the methodology, then efforts should have been made to ensure all the participants received these packs. Seed packs were given as an incentive to encourage farmers to buy their own after experiencing the production, harvesting and tasting of the crops during food preparation demonstrations, and learning about the nutritive value of these new crops. According to Sperling, seed system interventions can spur agricultural growth and often focus on accelerating the delivery of new varieties and good seeds [48]. Ojiewo also reported that limited access to quality seeds of improved varieties at affordable prices due to inadequate seed systems has reduced their contribution to improving nutrition and reducing poverty in East and West African regions [49].

An unexpected finding was that key informants indicated that a lack of money was a challenge affecting continued demand for the promoted/recommended dietary diversity. This finding was consistent with what interview participants also thought as household income was reported as a major determinant of household food consumption. This finding was contrary to statements by some key informants as well as many participants themselves who reported that one of the lessons learned from the intervention was that eating healthy should not cost a lot of money. The observation was however in line with some other researchers’ findings that household income is a major determining factor of the demand for not only food but other household commodities as well [50]. A vegetable seed pack costs less than US$1 in many agro-dealer shops in Zimbabwe and a seed pack is enough to produce vegetables that can be utilised by a family of up to six for more than a month. If participants were not prepared or willing to buy these seeds for themselves, following their learning of the benefits and need to diversify production and consumption, then this is a major impediment to increased demand for varied nutritious foods. Similarly, if the increased knowledge about food, nutrition, and the importance of diversifying diets is not enough to motivate people to invest in nutritious seeds to produce nutritious foods, then this creates a great threat to the sustainability of the food demand and consumption patterns observed in this study. Considerations in the context of the problem of sustainable food production is critical [51].

Key informants also reported a shortage of intervention staff to adequately support intervention participants and implement all the intervention activities as planned. This finding is contrary to other findings which indicated a perception by key informants and some participants in this study that poor food consumption and food demand behaviours by farmers were due to a lack of knowledge [52]. Continuous support is necessary to ensure sustained practising of the newly adopted behaviours.

Another observation from this study was that nearly all participants indicated that they were receiving some form of support, with the majority coming from NGOs. Unfortunately, the study did not ask the form or type of support, only the source was established. The assumption made by the researchers was that the reported support included the intervention. Food aid or any other external support to households affects the eating practices and behaviours of the concerned households [21]. This cannot, however, be expected to be sustainable, nor can it be used as an indication of an increase in demand for that type of food or the adoption of better eating practices. What happens post this support is what matters most.

This study also revealed that in addition to income, which was reported to be affecting both household and child-feeding practices significantly, other factors include the quantity of the harvest (household food availability and access) and family dynamics–including size and preferences [53]. It appears from these findings that agriculture production is a major determinant and hence an indicator of the food demands in the Makoni District [20]. This finding can be generalised to all rural communities that rely mostly on what they produce for their food needs. Similarly, a lack of agricultural inputs was revealed as another factor affecting food demand.

As far as children’s food consumption was concerned, children’s food preference was reported to be a major determinant of their consumption patterns. It appears mothers fed children what they liked more than what was healthy and beneficial to the child. It is worth noting that children’s palates and food preferences are developed from the foods that they are introduced to by caregivers [54]. Inappropriate foods introduced to children early during the initiation of complementary feeding have a bearing on what they choose and prefer to eat as they grow older. This finding further underscores the importance of caregivers learning about proper complementary feeding early, before they introduce the foods to children, so they do it appropriately when they start [55]. Timely introduction of the right type of complementary foods is essential for proper child growth and development [56].

An interesting finding of this study, which is contrary to other findings and beliefs [5, 47], was that culture was not a major factor affecting food consumption and food demand aspects promoted by the intervention. This could be explained by the fact that the intervention conducted a barrier analysis at the start and designed the behaviour communication change approach informed by the findings. In so doing targeted behaviours were not the culturally contentious ones but were contextualised. It could also be that the participants in this study do not have many problematic food consumption behaviours that could not be addressed by the behaviour communication change approach used. Culture affected mostly exclusive breastfeeding but tended to demystify some cultural practices of withholding certain foods from some family members such as women and children [57]. This finding is very important and empowering because ingrained cultural practices are in some contexts a major barrier to optimal nutrition particularly affecting food consumption [47, 58].

This study also found that parents had high hopes for prosperous futures for their children. Participants were also able to link malnutrition to poor development and cognitive capacity of children, in line with what other researchers have written [53, 59]. This finding can be used to actively promote healthy food consumption and demand for diverse nutritious foods to improve children’s nutritional status for better, well-developed futures.

Contrary to other research findings [60], this study revealed that the local markets had very little influence on the demand and consumption of diverse nutritious foods. Local markets were not the major sources of food for the intervention participants who relied mostly on what they produced. The market supply of foods was, however, determined by the participants’ demand. Participants only sought those foods they could not produce themselves from the local markets. It appears that these markets were the source of the high-calorie, low-nutritive value products that some children were consuming. Given this, if formal markets stocked nutritious foods and went a little further to market or advertise these foods, this has the potential to increase demand for these healthier options [61].

Farmer-to-farmer informal markets appeared stronger in determining food demand and consumption patterns [62]. Increased production of healthy, diversified crops and livestock in Makoni District is therefore likely to increase the demand for these foods by farmers within the district. For example, the study observed that egg consumption was low, and this could be because of low egg production by farmers in the area, although participants also mentioned that it was taboo for pregnant women to consume eggs. Unfortunately, the study did not look much into small livestock farming but concentrated more on crop production. This was one major limitation of the study.

## 4. Conclusions

The study findings of the evaluation of the intervention showed that nutrition behaviour change can be effective when delivered in a nutrition-sensitive agricultural intervention to influence the demand and consumption of diverse nutritious foods. Diversified agriculture production patterns in this study can be taken as reflective of the food demand, which in this case changed significantly because of the intervention. The intervention stimulated: i) an increase in demand for diverse nutritious foods, primarily through the structured and systematic ENIPPA methodology; ii) support for the production of a wide range of vegetables in small backyard gardens micro-gardens; and iii) the incorporation of other key complementary messages concerning hygiene promotion and childcare. It was anchored on a clear understanding of the nutritional problems in the Makoni District to be addressed by the intervention. The intervention clearly defined ten key behaviours to promote and conducted a barrier analysis to identify the impediments to practising these behaviours within the communities. This helped in designing clear and simple activities to target a change in these behaviours.

A few shortcomings of the intervention were identified. Key informants in this study reported low coverage of the intervention within the districts–this would have a negative bearing on the impacts at the district level. Intervention staff were few and therefore unable to fully support all participants effectively–this had a bearing on the success of the intervention. The vegetable seeds provided to the farmers to support the establishment of micro-gardens were few and could not reach all intervention participants. This caused disgruntlement among those who did not receive and hence affected their morale. These issues could have had a bearing on the sustainability of the intervention’s gains and could have been avoided through proper planning of the interventions.

The factors influencing the demand for varied and nutritious foods were many, with the major one being income. Participants and key informants in this study both indicated that income is necessary to support the production of varied foods and to buy other foods that could not be produced but were needed as part of diversified diets. Participants in this study also reported food preferences, household food availability, and food access as other key factors affecting their food consumption patterns. Culture did not seem to influence the demand and consumption of nutritious foods, instead, the intervention helped to demystify myths and promote healthy dietary patterns. Local formal markets did not influence food consumption. Their stocks were influenced by what was demanded by their customers. In this respect, there is scope that with some form of marketing and promotion they can influence the demand for nutritious foods locally.

## Supporting information

S1 Data Quantitative data. (XLSX)
